# Ultrasound Biomicroscopy Might Predict the Outcome of Phacoemulsification-Visco Dissection in Medically Controlled Primary Angle-Closure Glaucoma Eye With Extensive Peripheral Anterior Synechia

**DOI:** 10.3389/fmed.2021.705864

**Published:** 2021-07-19

**Authors:** Haijun Gong, Xu Dong, Bingru Zheng, Xinbo Gao, Liming Chen, Simin Zhang, Chengguo Zuo, Mingkai Lin

**Affiliations:** ^1^State Key Laboratory of Ophthalmology, Guangdong Provincial Key Laboratory of Ophthalmology and Visual Science, Zhongshan Ophthalmic Center, Sun Yat-sen University, Guangzhou, China; ^2^Guangdong Provincial Key Laboratory of Malignant Tumor Epigenetics and Gene Regulation, Department of Ophthalmology, Sun Yat-sen Memorial Hospital, Sun Yat-sen University, Guangzhou, China; ^3^The Fourth People's Hospital of Shenyang, Shenyang, China

**Keywords:** primary angle-closure glaucoma, ultrasound biomicroscopy, phacoemulsification, visco dissection, predictability

## Abstract

**Objective:** The treatment procedures of primary angle-closure glaucoma (PACG) with extensive peripheral anterior synechia (PAS) is a subject of debate. This study is to investigate the clinical features of phacoemulsification (Phaco)-visco dissection in medically controlled PACG patients with PAS > 180° and evaluate the predictability of Ultrasound Biomicroscopy (UBM) parameters on postoperative intraocular pressure (IOP).

**Methods:** 48 eyes (48 patients) with acute angle-closure glaucoma (AACG) and 30 eyes (30 patients) with chronic angle-closure glaucoma (CACG) were prospectively included. All patients underwent phaco-viscogoniolysis and foldable lens implantation and were followed for 1 year after surgery. We analyzed preoperative and postoperative IOP, the numbers of anti-glaucoma medicine, the visual field value, the extent of PAS, and UBM parameters alterations, and evaluated the correlation between preoperative UBM parameters and one-year postoperative IOP.

**Results:** IOP reduced significantly from 16.24 ± 4.33 to 14.49 ± 3.69 mmHg in AACG group and from 16.16 ± 3.69 mmHg to 14.31 ± 4.12 mmHg in CACG group, and PAS decreased significantly from 270 ± 60.33 to 171 ± 56.44° in AACG group and from 285 ± 70.46 to 168 ± 61.32° in CACG group, and the number of anti-glaucoma drugs decreased significantly in both groups. Several UBM parameters, including anterior chamber depth, trabecular iris angle, and peripheral iris thickness 500 increased while iris convex reduced considerably in the two groups, and angle opening distance 500 and trabecular-meshwork ciliary process angle increased significantly only in AACG group. One-year postoperative IOP correlated with preoperative angle opening distance 500 and trabecular iris angle negatively and iris convex positively.

**Conclusion:** Phaco-visco dissection can effectively reduce IOP, the numbers of glaucomatous medications, and PAS in medically controlled PACG patients with extensive PAS. UBM parameters might be valuable for analyzing postoperative anterior segment structure and predicting postoperative IOP of Phaco-visco dissection in PACG patients with extensive PAS.

## Introduction

Primary angle-closure glaucoma (PACG) is a leading cause of blindness in East Asia (1). East Asians have the well-known anatomic configurations, including a shallower anterior chamber depth (ACD), shorter axis length (AL), thicker lens, and smaller radius of the cornea than Westerners, thereby causing a higher prevalence of PACG (2). The classical clinical classification of PACG includes acute angle-closure glaucoma (AACG) and chronic angle-closure glaucoma (CACG), in which the anterior chamber angle can be permanently closed by peripheral anterior synechia (PAS). Previous studies reported that PACG patients with extensive PAS >180 degrees should be the candidates for filtering surgery because of their inefficient trabecular meshwork cellular capability and insufficient medical therapeutic effect (3). Although sole-filtering surgery or combined glaucoma and cataract surgery can cause a remarkable intraocular pressure (IOP) decrease, unfortunately, these procedures also bring about a high risk of short- and long-term complications, such as filtering bleb scarring and shallow anterior chamber (4).

Phacoemulsification-goniosynechialysis (PEGS) has been reported to have a lowering effect on IOP and reduce the use of antiglaucoma drugs in patients with medically uncontrolled (primary angle closure) PAC (5), AACG (6), medically controlled CACG (7), and primary open angle glaucoma (POAG) (8). Evidence suggested cataract extraction by phacoemulsification (Phaco) can result in an IOP decrease due to its effects including, but not limited to, ultrasound on the trabecular meshwork cellular, mechanical washout on the trabecular meshwork, and uveal tract outflow (9). Additionally, tearing off PAS from the anterior chamber angle wall by PEGS is an effective procedure to reestablish outflow structure and increase drainage of aqueous humor (10). Our clinical work used an alternative safe and minor procedure—combined Phaco and visco dissection in some medical controlled PACG patients with PAS and observed a relatively long-term controlled IOP. It indicated medically controlled PACG eye with PAS >180° might have remaining aqueous outflow function and can also be the candidates for combined Phaco and visco dissection. However, there is limited information related to the clinical outcome and the preoperative predictors in these patients. Ultrasound biomicroscopy (UBM) is a non-invasive imaging technique and widely be used to assess the morphology changes of glaucoma and measure the anterior segment structure. Interestingly, numerous studies have shown UBM parameters changes and have diagnostic capabilities following cataract surgery in patients with glaucoma (11–13). Therefore, evaluating the quantitative changes of preoperative and postoperative characters and understanding the link between UBM parameters and clinical features in PACG with extensive PAS might help ophthalmologists manage this disease.

In this study, we analyzed the effect of combined Phaco-visco dissection on IOP, numbers of antiglaucoma drops, and PAS degrees in PACG patients with PAS >180° and IOP controlled before surgery. Meanwhile, we used UBM to detect the morphologic changes of anterior segment parameters and hopefully explored their prediction roles in postoperative IOP.

## Methods

### Participants

This prospective case series evaluated patients between January 2018 and December 2019 at the Zhongshan Ophthalmic Center, Sun Yat-sen University. This study was approved by the medical ethics committee of Zhongshan Ophthalmic Center and was performed following the Declaration of Helsinki's tenets.

This study evaluated 78 eyes of 78 patients, including 48 AACG patients and 30 CACG patients. The AACG patients had an elevated IOP history (acute, subacute, and intermediate attacks) and symptoms remission before inclusion. The time between controlling and attack of AACG ranged from 1 month to 2 months. At the time of surgery, no change in glaucoma medication administration and visual field parameters and target IOP < 21 mmHg in the last 1 month was considered a medically controlled condition in AACG and CACG group. In both groups, each patient had preoperative gonioscopy showing PAS >180° and had a typical visual field and optical nerve defects, coexisting cataract, and the best corrected visual acuity (BCVA) <0.5 (decimal). In two eyes of each participant, we selected the eye with more severe PAS. The exclusion criteria were having a history of intraocular surgery, laser iridotomy, extra non-glaucoma laser conditions, ocular trauma, intraocular inflammation, ischemic disease, and secondary glaucoma histories such as vascular glaucoma, pseudoexfoliation, pigmentary glaucoma, glaucomatocyclitic syndrome, lens, and corticosteroid-induced glaucoma.

### Examination and Surgical Procedure

**Figure 1:**
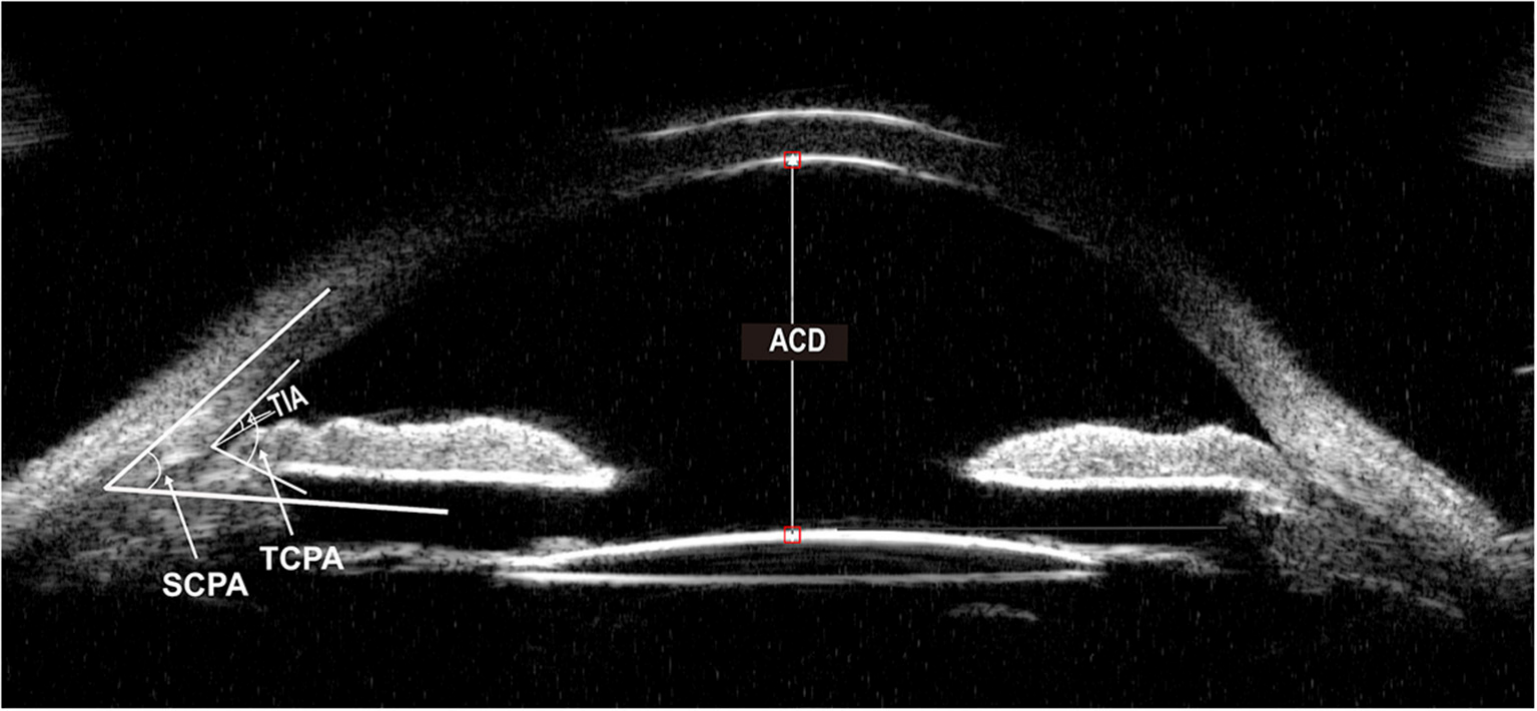
Ultrasound biomicroscopic (UBM) image of ACD, TIA, TCPA, SCPA. ACD, anterior chamber depth. TIA, trabecular iris angle. TCPA, trabecular-meshwork ciliary process angle. SCPA, scleral ciliary process angle.

**Figure 2:**
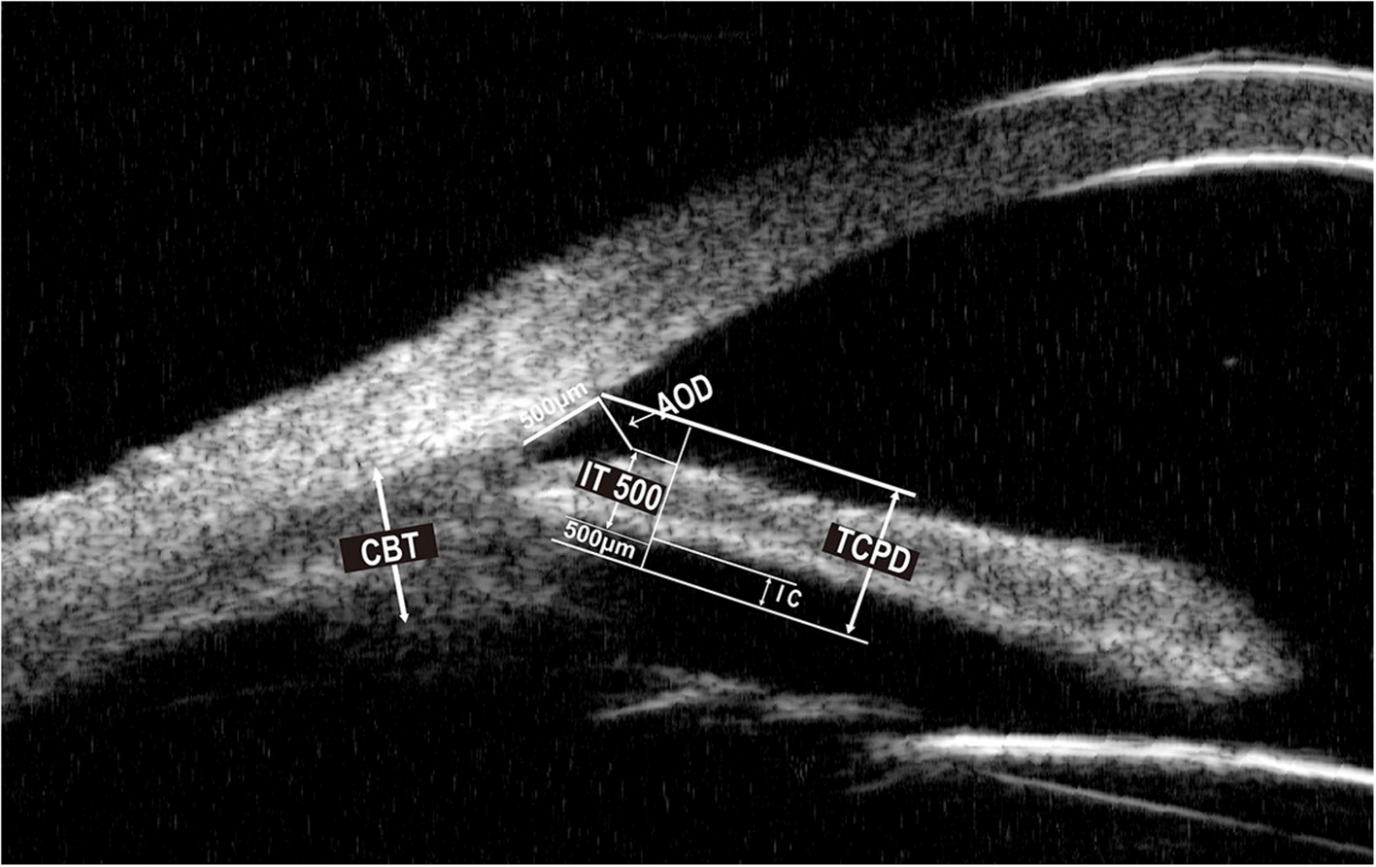
Ultrasound biomicroscopic (UBM) image of AOD500, TCPD, IT500, CBT, IC. AOD500, angle opening distance 500. TCPD, trabecular ciliary process distance. IT500, peripheral iris thickness 500. CBT, ciliary body thickness. IC, iris convex.

Baseline parameters were evaluated 1 week before surgery, and the follow-up examinations were 1 day, 1 week, 1 month, and then every 3 months till 1 year. Baseline demographics, the number of medications before and after the operation, visual acuity testing, IOP, MD (mean deviation) value of the visual field, slit-lamp examination, and ophthalmoscopy results were recorded. Angle structure was evaluated with a Zeiss four-mirror gonio lens. The PAS degree was evaluated by senior glaucoma subspecialists and confirmed by the same surgeon to keep the examination consistency. The visual field was measured by automated program 24-2 threshold perimetry (Humphrey 750i, Zeiss, Germany). UBM examinations were performed 1 week before surgery and 1 month, 6 months, and 1 year after surgery. Anterior parameters including ACD, angle-opening distance 500 (AOD500), scleral ciliary process angle (SCPA), trabecular-meshwork ciliary process angle (TCPA), trabecular ciliary process distance (TCPD), trabecular iris angle (TIA), peripheral iris thickness 500 (IT500), ciliary body thickness (CBT), and iris convex (IC) were obtained using the UBM technique (SW-3200L, Suoer, China). Figures 1, 2 illustrated their calculation method: ACD was the length from the front lens surface to the corneal endothelium; AOD500 was defined as the distance between the point of the posterior corneal surface at 500 mm from the scleral spur and the anterior iris surface; TCPD has defined the perpendicular distance between a point spreading out from scleral spur to the corneal endothelium at 500 mm and the ciliary processes; CBT was detected at 1 mm posterior from the scleral spur; TIA was the angle passing through a point on the corneal endothelium at 500 mm from the scleral spur and the point on the iris vertically opposite, and it represents the open degrees of anterior angle; SCPA was the angle between the tangent line to the scleral surface and the axis of the ciliary processes, and it represents the ciliary body location; TCPA was defined as the angle between the line on corneal endothelium at 500 mm from the scleral spur and the front surface of ciliary processes; IT500 was detected from the iris point 500 mm and represented iris thickness and location; IC was measured as the perpendicular length between iris apex and the lowest point of pupillary margin to the iris rear surface's highest point.

All surgeries were performed by one surgeon who used the same procedure. It was performed by topical anesthesia, 3-mm corneal incision, continuous circular capsulorhexis using a 27-gauge bent needle, and standard phacoemulsification. A single-piece acrylic IOL (SA60AT, Alcon, US) was inserted into the posterior chamber through the corneal incision. According to preoperative records, viscoelastic material was used to strip the most peripheral iris from angle adhesion positions bluntly after lens implantation. Then we used an irrigation-aspiration system to remove the remaining viscoelastic material from the anterior chamber. After surgery, all patients were treated with topical corticosteroids, antibiotics, and antiglaucoma medications. Topical antibiotics and steroids were trapped within 6 weeks. All patients were followed 1 day, 1 week, 2 weeks, 1 month, and then every 3 months till 1 year after surgery. To evaluate the long-term IOP and PAS reduction, we only compare the preoperative and 1-year postoperative parameters.

### Statistics Methods

SPSS version 21.0 for Social Sciences (IBM SPSS Inc., NY, US) was used for statistical analysis. All numerical data were expressed as mean ± SD, and the Student *t*-test was performed to compare all parameter variations from baseline. Gender and laterality differences were assessed by Chi-square test. Pearson correlation was used to study the correlation between 1-year postoperative IOP and preoperative UBM characteristics. A *p*-value < 0.05 was considered statistically significant.

## Results

Table 1 provides the baseline characteristics of all 78 participants. The mean age of subjects was 68.07 ± 3.97 years in the AACG group and 66.87 ± 2.38 years in the CACG group. There were no statistically significant differences in all demographic features and preoperative visual acuity and MD value between the AACG and CACG groups.

**Table 1:** Patients characteristics.

	**AACG (*n* = 48)**	**CACG (*n* = 30)**	***P***
Age (years)	68.07 ± 3.97	66.87 ± 2.38	0.139
Sex (M: F)	19:29	12:18	0.971
Laterality (OD: OS)	26:22	13:17	0.352
Preoperative BCVA (decimal)	0.37 ± 0.13	0.43 ± 0.12	0.071
Preoperative MD (dB)	−17.59 ± 10.16	−15.23 ± 12.20	0.231
Range	−29.62 to −2.36	−28.43 to −1.19	

*BCVA, best corrected visual acuity; MD, mean deviation*.

Table 2 shows the clinical features of preoperative and 1-year postoperative stages, including IOP, numbers of lowering IOP eye drops and, the extent of PAS and MD value in the two groups. IOP decreased significantly in the two groups, from 16.24 ± 4.33 to 14.2 ± 3.6 mmHg in the AACG group and from 16.16 ± 3.69 to 14.31 ± 4.12 mmHg in the CACG group. The PAS degree also reduced remarkably in both groups, from 270 ± 60.33 to 171 ± 56.44° in the AACG group and from 285 ± 70.46 to 168 ± 61.32° in the CACG group. Meanwhile, both groups had a meaningful decrease in numbers of antiglaucoma drugs, from 1.74 ± 1.46 to 1.12 ± 0.94 in the AACG group and from 2.23 ± 1.54 to 1.20 ± 0.76 in the CACG group. There was also a significant difference in the numbers of postoperative antiglaucoma drugs between the AACG and the CACG group. However, the MD value did not differ significantly in both groups.

**Table 2:** The changes of preoperative and 1-year postoperative clinical parameters.

	**Preoperative value**	**Postoperative value**	***P***
**IOP (mmHg)**			
AACG	16.24 ± 4.33	14.49 ± 3.69	0.012^*^
CACG	16.16 ± 3.69	14.31 ± 4.12	0.009^*^
*P*	0.519	0.104	
**NO. Eye drop (*****n*****)**			
AACG	1.74 ± 1.46	1.12 ± 0.94	0.007^*^
CACG	2.23 ± 1.54	1.20 ± 0.76	0.001^*^
*P*	0.274	0.035^*^	
**Degree of PAS (degrees)**			
AACG	270 ± 60.33	171 ± 56.44	<0.001^*^
CACG	285 ± 70.46	168 ± 61.32	<0.001^*^
*P*	0.402	0.595	
**MD (dB)**			
AACG	−17.59 ± 10.16	−16.34 ± 11.03	0.381
CACG	−15.23 ± 12.20	−14.15 ± 11.65	0.912
*P*	0.354	0.547	

* *Means p < 0.05. IOP, intraocular pressure; PAS, peripheral anterior synechia; MD, mean deviation*.

Table 3 provides the changes of UMB parameters. Each value in the four positions (12, 3, 6, and 9 o'clock) was measured, and their mean value was selected for analysis. The increase in ACD, TIA, and IT500 and decrease in IC were statistically significant in both groups, whereas AOD500 and TCPA just increased significantly within the AACG group only following the surgery. In the meanwhile, compared with the CACG group, the AACG group had a lower postoperative value in AOD500, TIA, and IC.

**Table 3:** The changes of preoperative and 1-year postoperative UBM parameters.

**UBM parameters**	**Preoperative value**	**Postoperative value**	***P***
**ACD (mm)**			
AACG	1.77 ± 0.31	3.33 ± 0.55	<0.001^*^
CACG	1.78 ± 0.23	3.21 ± 0.58	<0.001^*^
*P*	0.345	0.523	
**AOD500 (mm)**			
AACG	0.012 ± 0.022	0.075 ± 0.059	<0.001^*^
CACG	0.0785 ± 0.0616	0.1882 ± 0.6977	0.610
*P*	0.112	0.002^*^	
**TIA (degrees)**			
AACG	1.168 ± 2.266	7.684 ± 6.226	<0.001^*^
CACG	1.6390 ± 1.5588	8.0773 ± 6.4486	0.001^*^
*P*	0.124	0.016^*^	
**TCPD (mm)**			
AACG	0.4639 ± 0.1167	0.5235 ± 0.1152	0.120
CACG	0.4616 ± 0.1182	0.5348 ± 0.1096	0.121
*P*	0.234	0.315	
**SPCA (degrees)**			
AACG	37.7347 ± 4.721	39.7911 ± 2.8719	0.128
CACG	38.5801 ± 4.3325	39.2944 ± 2.7639	0.668
*P*	0.345	0.521	
**TCPA (degrees)**			
AACG	46.6730 ± 9.8928	56.7547 ± 10.6560	0.004^*^
CACG	49.2000 ± 11.2269	56.6045 ± 10.5147	0.097
*P*	0.081	0.124	
**IT500(mm)**			
AACG	0.3635 ± 0.0615	0.4404 ± 0.0652	<0.001^*^
CACG	0.3516 ± 0.0709	0.4357 ± 0.0936	0.018^*^
*P*	0.106	0.539	
**CBT (mm)**			
AACG	0.6878 ± 0.1313	0.7193 ± 0.1241	0.452
CACG	0.6922 ± 0.1641	0.7087 ± 0.1627	0.802
*P*	0.775	0.732	
**IC (mm)**			
AACG	0.2304 ± 0.1034	0.0969 ± 0.0819	<0.001^*^
CACG	0.6953 ± 0.1732	0.1838 ± 0.3945	<0.001^*^
*P*	0.007^*^	0.003^*^	

* *Means p < 0.05. ACD, anterior chamber depth; AOD500, angle opening distance 500; TIA, trabecular iris angle; TCPD, trabecular ciliary process distance; SCPA, scleral ciliary process angle; TCPA, trabecular-meshwork ciliary process angle; IT500, peripheral iris thickness 500; CBT, ciliary body thickness; IC, iris convex*.

Table 4 shows the correlation between 1-year postoperative IOP and preoperative UBM parameters in two groups. Postoperative IOP was negatively correlated with preoperative AOD (*R* = −0.383, *p* = 0.043) and TIA (*R* = −0.383, *p* = 0.043), and related to preoperative IC positively (*R* = 0.557, *p* = 0.003). There was also a significant correlation between preoperative IOP and 1-year postoperative in both CACG group (*R* = 0.475, *p* = 0.015) and AACG group (*R* = 0.442, *p* = 0.023).

**Table 4:** Correlation coefficients between preoperative UBM parameters and 1-year postoperative IOP in two groups.

**Variables**	**AACG**		**CACG**	
	**R**	***P***	**R**	***P***
ACD	0.205	0.187	−0.071	0.440
AOD500	−0.383	0.043^*^	0.450	0.155
TIA	−0.383	0.043^*^	−0.450	0.155
TCPD	−0.320	0.078	0.000	0.500
SPCA	0.141	0.272	0.143	0.380
TCPA	−0.294	0.098	−0.179	0.351
IT500	0.333	0.070	−0.036	0.470
CBT	0.058	0.402	0.214	0.322
IC	0.577	0.033^*^	0.179	0.351

* *Means p < 0.05. ACD, anterior chamber depth; AOD500, angle opening distance 500; TIA, trabecular iris angle; TCPD, trabecular ciliary process distance; SCPA, scleral ciliary process angle; TCPA, trabecular-meshwork ciliary process angle; IT500, peripheral iris thickness 500; CBT, ciliary body thickness; IC, iris convex*.

**Figure 3:**
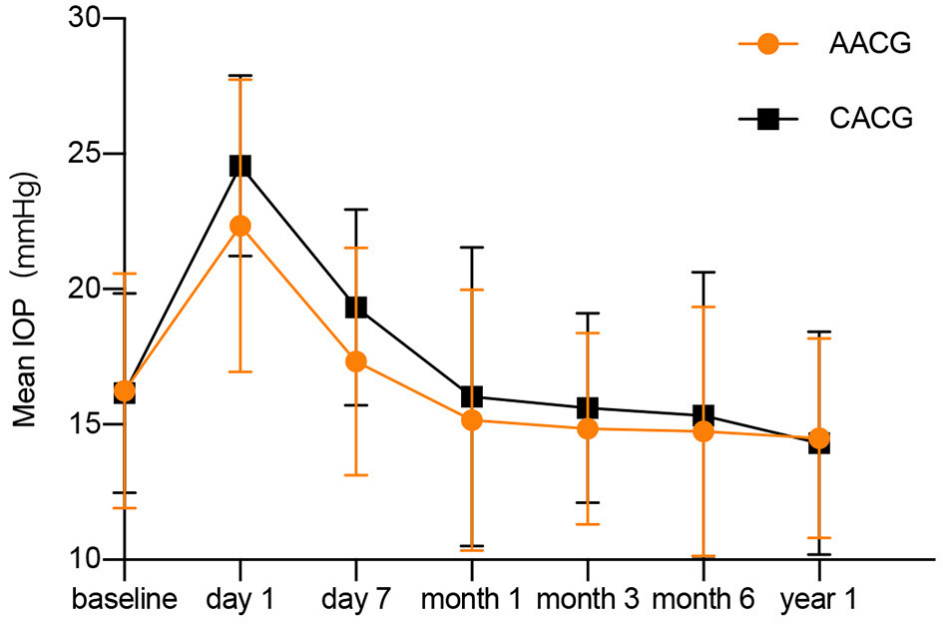
The changes of intraocular pressure after surgery in acute angle-closure glaucoma and chronic angle-closure glaucoma patients.

For the 1-year follow-up period, none of the patients in both groups has an IOP spikes history (Figure 3). Assessment of intraoperative complications showed that two patients in the AACG group and three patients in the CACG group experienced intraoperative anterior chamber hemorrhage, but it didn't affect the final vision and IOP. None of the participants in both groups experienced severe complications within 1 year after surgery. Postoperative re-PAS occurred in six cases (12.5%) in the AACG group and seven cases (23.3%) in the CACG group, and the major sites were identical to the preoperative site but didn't require further intervention.

## Discussion

In this study, we first found IOP, degree of PAS, and anti-glaucoma medication decreased significantly in both AACG and CACG groups after Phaco-visco dissection surgery. According to PAS degrees, regarding numbers of antiglaucoma medication, and the condition of visual acuity and cataract severity, the surgical approach of PACG with PAS may be a subject of debate. Surgical treatment approaches in those patients include phacoemulsification, trabeculectomy, Phaco-trabeculectomy, goniosynechialysis, and Phaco-goniosynechialysis (14). A meta-analysis of angle-closure glaucoma reported that IOP in the PEGS group decreased significantly more than in Phaco alone group, and there was no statistical difference between PEGS group and Phaco-trabeculectomy/ trabeculectomy group (15). This is in accordance with our study that Phaco-visco dissection has a satisfactory IOP decrease and a possible improvement of trabecular meshwork function in both groups. Therefore, using this lower-risk and simpler procedure rather than filtering surgery to restore the normal anterior chamber anatomic structure and clear the aqueous outflow from any obstruction will be appealing for those medically controlled patients with extensive PAS.

UBM is the most useful method of imagining anterior segment configurations and their clinic implications and evaluating the result of PACG treatment (16). Previous UBM studies showed these eyes have shorter TCPD and anteriorly positioned ciliary process (17). Sang Woo Park reported that the ACD, AOD500, TIA, TCPD, and SCPA increased significantly after the glaucoma triple procedure (18). The ciliary process position can directly influence TCPD. Nonaka reported, after Phaco surgery, ACD, AOD500, and TCPD increased due to widening of the angle, removal of the pupillary block, and reduction in the anterior position of the ciliary processes (19). Our study found similar results that ACD, TIA, and IT500 increased in both groups and AOD500 and TCPA just increased significantly in the AACG group following the surgery. The TCPD was also increased but had no statistical significance in both groups. We also found postoperative IOP was negatively associated with preoperative AOD and TIA and positively related to preoperative IC. It means higher preoperative AOD and TIA may easily lead to lower postoperative IOP, whereas thicker IC might cause higher postoperative IOP. We suppose it is because high AOD and TIA may induce a low incidence of anterior chamber angle-closure, but IC may represent a high pupillary block risk. These parameters may also hopefully be the predictors for the postoperative IOP accordingly when making the surgery decision.

This prospective study has several limitations. Small-scale and all participants from the same race in China is the principal limitation. To further verify our results and obtain stronger evidence of Phaco-visco dissection effects on those patients, large-scale randomized controlled trials are necessary for the prospective study. In addition, the clinical and UBM data for multiple time points should be included and assessed in the future study to provide more detailed information, such as long-term re-PAS rate and IOP level, thereby assisting clinicians regarding PACG patients' postoperative treatment and management.

In conclusion, we have shown that combined Phaco and visco dissection has an advantage in lowering IOP, decreasing numbers of antiglaucoma drops, and ameliorating anterior chamber angle structure and function in PACG patients with extensive PAS degree. UBM parameters may be possible predictors for the effect of Phaco and visco dissection on postoperative IOP.

## Data Availability Statement

The raw data supporting the conclusions of this article will be made available by the authors, without undue reservation.

## Ethics Statement

The studies involving human participants were reviewed and approved by the medical ethics committee of Zhongshan Ophthalmic Center. The patients/participants provided their written informed consent to participate in this study.

## Author Contributions

ML, XD, and HG designed this study. BZ and XG collected and measured data. LC and SZ analyzed data. CZ and HG wrote this article and revised the manuscript. All authors discussed the results and commented on the manuscript.

## Conflict of Interest

The authors declare that the research was conducted in the absence of any commercial or financial relationships that could be construed as a potential conflict of interest.
